# Degradation of atrazine and bromacil in two forestry waste products

**DOI:** 10.1038/s41598-021-83052-z

**Published:** 2021-02-08

**Authors:** Trevor K. James, Hossein Ghanizadeh, Kerry C. Harrington, Nanthi S. Bolan

**Affiliations:** 1 AgResearch, Ruakura Research Centre, Private Bag 3123, Hamilton, 3240 New Zealand; 2 School of Agriculture and Environment, Massey University, Private Bag 11-222, Palmerston North, 4442 New Zealand; 3 Global Centre for Environmental Remediation, ATC Building, University of Newcastle, Callaghan, NSW 2308 Australia

**Keywords:** Pollution remediation, Environmental impact

## Abstract

The persistence and degradation of two common herbicides, atrazine and bromacil in two organic media, wood pulp and sawdust were compared with two soils. The hypothesis tested was that herbicide degradation will be faster in high organic matter media compared to soil. Degradation of two herbicides was carried out in four different temperature regimes and in sterilised media. The degradation half-life (t½) was determined under above-mentioned conditions then compared to degradation in soil. The degradation as quantified by t½ of the herbicides was generally longer in both organic media. Although microbial degradation was an important factor in the mineralisation of these herbicides, overall, the pH of the media had a more profound effect on the desorption and subsequent degradation rate than the organic carbon content. The results of this study revealed that the hypothesis was only partially correct as organic matter content per se did not strongly relate to degradation rates which were mainly governed by pH and microbial activity.

## Introduction

The performance of residual herbicides is strongly influenced by the soil^1^. Several soil parameters are known to affect herbicide performance including the pH of the soil solution, soil structure, sand content and type of clay^2^. However, the single most influential soil property is the soil organic carbon^3,4^. The two main influences of soil organic carbon are its ability to adsorb the active pesticide molecules, thus rendering them biologically inactive^5^, and as a host for soil microbes which generally are the principal route for herbicide degradation^6,7^.

The "Rich Ditch" system uses a furrow filled with forestry waste such as sawdust or wood pulp (foreign growing media) to support the crop while nutrients are supplied hydroponically^8^. The concept was to use the system to grow one or two high value crops hydroponically and afterwards the partly decomposed waste material used in the ditch would be incorporated into the soil and a traditional crop grown^8^. The “Rich Ditch” system has the potential to reduce herbicide input as the waste material used in the ditch contains no weed seeds so fewer herbicides would be required in the vicinity of the sensitive plants^8^. However, the area between the rows remains partially exposed or thinly covered soil and thus is the source of weed seeds and subsequently weed growth. These weeds could be controlled mechanically with cultivation, but it would be more cost effective to control them with a banded herbicide application.

The “Rich Ditch” system introduces large quantities of organic carbon into the immediate environment of herbicides used in the inter-row areas, and therefore it is highly likely to have a significant impact on the behaviour of these herbicides. One of the areas of herbicide behaviour that can be noticeably affected by the introduction of foreign growing media is the persistence and degradation of applied herbicides^9^. The persistence of herbicides in soil is critical to developing their environmental profile and eventual fate^10,11^. The degradation rates of herbicides in soils can be thought of in terms of the molecules intrinsic stability to microbial and chemical decay, which is modified by soil factors such as organic matter, pH and climate^12^. In soils with a high organic matter content (generally > 10%), the primary factor controlling herbicide degradation in soil is microbial transformation^13^. This degradation often follows a simple exponential decay model and is thus assumed to follow first-order kinetics^14,15^. Degradation in soils with high organic matter and adequate rainfall tends to be much faster than in soils with low organic matter or where moisture is a limiting factor, due to the higher levels of microbial activity in the high organic matter soils^10^.

Carrying out degradation and persistence studies in the field will produce an environmental profile that cannot be refuted as it arguably fits a real scenario^16^. However, it is difficult to quantify and parameterise the various environmental conditions that influence degradation in such field situations as they are constantly changing. Without an adequate understanding of the interactions with key environmental conditions it then becomes very difficult to extrapolate and predict herbicide degradation under diverse conditions^17^. It is easier and more economical to determine the effect of environmental parameters, such as temperature and microbial activity, on degradation rates under controlled conditions and then extrapolate to field conditions^18^.

Atrazine and bromacil are two commonly used residual herbicides in New Zealand horticulture and could be used for controlling weeds associated with the “Rich Ditch” cropping system, atrazine for sweetcorn^19^ and bromacil for asparagus^20^. Both atrazine and bromacil are water soluble (33 mg/L and 815 mg/L respectively) with potential for leaching out of the plant growth zone. Atrazine has been reported to have a range of environmental impacts^21,22^. Bromacil causes environmental concerns due to its solubility and persistence^23^.

The objective of study outline in this manuscript was to determine the degradation parameters of atrazine and bromacil under a variety of environmental conditions in four diverse growing media, viz. Horotiu and Mangateretere soils, wood pulp and sawdust. The hypothesis being tested was that herbicides degrade more rapidly in the presence of high organic matter soil amendments. The experiments reported in this manuscript were carried out at three different temperatures which span the range of soil temperatures experienced in the field during the spring/summer growing season. A sterilised control was included to evaluate the importance of microbial activity on degradation. In addition, the influence of a fluctuating soil temperature regime on degradation^24^, was investigated because this has been found to enhance biological activity^25^.

## Materials and methods

### Media characteristic

The four media used in this study; sawdust, wood pulp and Mangateretere and Horotiu soils, were the same as those used by James et al.^26^. All studies were carried out at a constant water content of 60% maximum water holding capacity (MWHC). Several previous studies have showed little effect of soil moisture contents between 40 and 80% MWHC on degradation rates^10,27^. The MWHC was determined by the 24-h water saturation method using Hilgard cups^28^ (Table 1). The pH values of the four media were determined at the commencement of the degradation study using the slurry method^29^ (Table 1).

**Table 1:** Maximum water holding capacity values and pH the four media measured prior to commencement of the degradation experiments.

	Media			
	Horotiu soil	Mangateretere soil	Sawdust	Wood pulp
MWHC (%)	106	54	452	281
pH	5.51	5.44	5.59	7.71

### Degradation studies

Degradation studies were carried out in all four media for the herbicides, atrazine and bromacil. These two herbicides were selected as they are known to persist in the environment^30,31^, and would thus provide a good comparison between the media. The degradation was determined at a herbicide concentration in soil equivalent to a field application of twice the label use rate^32^, incorporated to a depth of 100 mm. The twice label rate was used as this is the worst case scenario that arises when spray overlap occurs during application in the field^33^. The studies were carried out at 10 °C, 20 °C, 30 °C and 20 ± 5 °C (as a diurnal sine curve) in the dark at 60% MWHC. Additionally, the 20 °C study was also carried out using autoclave-sterilised media^34^. The temperatures chosen are reasonable temperature extremes and approximate average temperature for the time of year the herbicides are likely to be used in New Zealand.

The moisture contents of the media were determined gravimetrically by drying quadruplicate samples (10 g) at 105 °C for 24 h. The media were then weighed into 250 mL Erlenmeyer flasks (10, 20 and 50 g dry wt equivalents for sawdust, wood pulp and the two soils, respectively) and brought to 2 g below the 60% MWHC weight by adding deionised water. Flasks for each herbicide were fortified by adding the required amount of herbicide in 2 mL of water and shaken by hand to mix the herbicide and media. Four additional aliquots of 2 mL were placed directly into small glass vials, sealed and frozen, to be used as controls. The herbicide fortification solution was made up from the formulated products Atradex (900 g kg^−1^ atrazine) and Hyvar X (800 g L^−1^ bromacil) to better match the field situation. The flasks were loosely sealed with aluminium foil and placed in the respective temperature regimes. They were weighed at weekly intervals and water added as required to maintain the moisture content. Eight flasks were frozen immediately after shaking (Day 0) and two were removed from each temperature regime and frozen after 3, 7, 14, 21, 28, 42, 56 and 84 days had elapsed. All flasks remained frozen at − 20 °C until required for analysis.

### Herbicide extraction

#### Procedure for atrazine extraction from media

The extraction method was a slight modification of James et al.^30^. After the flasks containing atrazine were defrosted, deionised water was added to bring the weight of water in the flask (including soil water) to 30 g and 70 ml of methanol was added. The flasks were sealed and shaken on an orbital shaker at 50 °C for 3 h and then allowed to settle overnight at ambient laboratory temperature (20 °C). If there was more than 30 g water present in the flask on completion of the study, then sufficient methanol was added to maintain a 70:30 methanol to soil water ratio. After settling, either a 10 ml aliquot of supernatant liquid was drawn off or, where more than 70 mL of methanol was added, then an aliquot equal to 10% of the total solution was drawn off. The aliquot was placed in a Schott bottle and 90 mL deionised water added. This was aspirated under vacuum through Extract-Clean solid phase extraction columns containing 0.5 g of C18 silica-based sorbent material. The sorbed material was eluted twice with 2 mL of methanol and the combined eluent evaporated to dryness under a gentle stream of nitrogen at 30 °C. The residue was re-dissolved in 0.5 ml of methanol and 0.5 ml of water added. The re-solubilised extract was filtered through an Anatop 10, 0.2 µm PTFE syringe filter in preparation for quantification by HPLC.

#### Procedure for bromacil extraction from media

The bromacil extraction method was modified from James and Lauren^35^. After the flasks containing bromacil were defrosted, deionised water was added to bring the weight of water in the flask (including soil water) to 30 g and 70 ml of 1.5% w/v aqueous sodium hydroxide was added. The flasks were sealed and shaken on an orbital shaker for 1 h at 20 °C and then allowed to settle overnight at ambient laboratory temperature (20 °C). If there was more than 30 g water present in the flask on completion of the study, then sufficient sodium hydroxide solution was added to maintain a 70:30 sodium hydroxide solution to soil water ratio. After settling, either a 10 ml aliquot of supernatant liquid drawn off or, where more than 70 mL of sodium hydroxide solution was added, then an aliquot equal to 10% of the total solution was drawn off. The aliquot was placed in a test tube and acidified to a pH of about 2–3 with 1 ml of 5 N hydrochloric acid. This was extracted with 10 mL of dichloromethane. After the dichloromethane was added it was vortex-mixed for 1 min and centrifuged at 3600 rpm for 10 min before the dichloromethane was drawn off. The volume of recovered dichloromethane was measured and then evaporated to dryness under a gentle stream of nitrogen at 30 °C. The residue was re-dissolved in 0.25 ml of methanol and 0.75 ml of water added. The re-solubilised extract was centrifuged at 2000 g for 20 min and filtered through an Anatop 10, 0.2 µm syringe filter in preparation for quantification by HPLC. Recoveries of atrazine and bromacil from the eight Day-0 samples are presented in Table 2 and percent recovery and standard error of the mean (SEM) for each combination are presented in Table 3. These data show that recovery from the high organic matter wood pulp and sawdust was both efficient (% recovery > 90%) and consistent (SEM < 2).

**Table 2:** Recovery of atrazine and bromacil from the eight Day-0 samples compared to the original fortification.

Media	Atrazine			Bromacil		
	Fortification	Recovery		Fortification	Recovery	
	(µg)	%	SEM	(µg)	%	SEM
Horotiu soil	207	96.4	3.01	457	85.7	0.85
Mangateretere soil	219	94.8	2.72	387	73.9	1.52
Wood pulp	307	96.2	0.96	503	94.9	1.66
Sawdust	281	93.1	1.98	492	99.3	1.90

**Table 3:** Degradation half-life (t_½_ (d) ± SE, assuming 1st order kinetics) and R^2^ linear regression statistic for degradation of atrazine and bromacil in four media under five different degradation regimes.

Media	Degradation temperature					
	10 °C		20 °C		30 °C	
	t_½_ (d)	R^2^ %	t_½_ (d)	R^2^ %	t_½_ (d)	R^2^ %
*Atrazine*						
Horotiu	17 ± 2	91.3	13 ± 3	76.7	12 ± 3	71.9
Mangateretere	89 ± 9	94.0	21 ± 1	98.3	17 ± 1	97.8
Wood pulp	125 ± 13	93.5	70 ± 5	96.0	39 ± 3	96.0
Sawdust	75 ± 6	95.5	39 ± 2	97.6	23 ± 2	96.4
*Bromacil*						
Horotiu	219 ± 47	75.2	136 ± 31	73.3	85 ± 13	86.0
Mangateretere	338 ± 121	53.0	485 ± 197	46.5	300 ± 67	74.1
Wood pulp	170 ± 24	88.1	64 ± 3	98.6	33 ± 2	97.2
Sawdust	724 ± 401	31.8	155 ± 11	87.9	68 ± 7	93.2

	Degradation temperature					
	20 ± 5 °C		20 °C sterile			
Media	t_½_ (d)	R^2^%	t_½_ (d)	R^2^%		
*Atrazine*						
Horotiu	13 ± 3	75.6	55 ± 4	95.9		
Mangateretere	21 ± 1	96.9	53 ± 4	95.9		
Wood pulp	79 ± 6	95.9	75 ± 8	92.0		
Sawdust	43 ± 5	92.2	45 ± 3	97.5		
*Bromacil*						
Horotiu	141 ± 26	81.0	153 ± 20	89.5		
Mangateretere	423 ± 267	26.5	418 ± 160	49.4		
Wood pulp	52 ± 3	97.7	288 ± 38	88.9		
Sawdust	183 ± 54	61.8	827 ± 475	30.2		

Generally, the herbicide recovery from soil was less efficient than wood pulp and sawdust Table 2). The results for bromacil recovery from soil are consistent with those of Yolliffe et al.^36^ who, using a similar method, recovered between 39 and 75% of added bromacil after 3 days in six different soils and 87% from a quartz sand. Zimdahl et al.^37^, also using the method of Yolliffe et al.^36^, averaged 87% recovery of bromacil. Furtula and Kuo^38^ obtained 80–88% recovery from soil following a shaking with methanol extraction of fortified samples. Strongly alkaline conditions are known to degrade substituted uracils^39^, but there is no reason to suspect that alkaline conditions would only do this in soil and not in either the wood pulp or sawdust so it was concluded that this was not a factor. Yolliffe et al.^36^ also found no evidence of alkaline degradation in the extraction procedure for up to 2 h.

Although the extraction of atrazine from the Horotiu and Mangateretere soils was more variable than from either the wood pulp or sawdust (Table 3), it was still in keeping with published values using a variety of extraction methods. For example, in a comparison of atrazine extraction by shaking with methanol or by accelerated solvent extraction (where methanol is pumped through the soil at elevated pressure and temperature) the SEMs for four soils (six replicates) ranged from 1.10 to 3.17% for the shaking method and from 0.35 to 3.5% for the accelerated solvent extraction^40^. The SEM for duplicate samples and using methanol:water (9:1) for extraction of atrazine from soil in a column leaching experiment ranged from 14 to 64%^41^. Using a similar method but with three replicates, Khan & Marriage^42^ recovered between 92.5 and 96.5% of atrazine from fortified soil samples with SEM values of 2.4 and 1.5 respectively. In a method using microwave assisted extraction (extracting directly into water at elevated temperature and pressure), Xiong et al.^43^ produced recovery data with SEMs ranging from 1.6 to 2.0% from three replicates.

The extraction and clean-up procedures proved effective for quantification by HPLC with no co-extractants adjacent to or interfering with the peak of the analyte. Sawdust contains many water soluble extractives (mostly polyphenols)^44^ and the methanol extraction of atrazine from sawdust concentrated highly coloured tannins^45^. However, these pigments eluted early from the HPLC column and resulted in little, if any, interference with the atrazine peak (data not shown). The sodium hydroxide extraction of bromacil from the wood pulp and sawdust also contained a significantly higher level of co-extractants than for either of the two soils, but they eluted rapidly through the HPLC column and did not result in any interference with the bromacil peak.

### Treatment of the control fortification samples

The control fortification samples were diluted in the scintillation vial with either 14 or 18 mL of purified water to give either an 8- fold or a tenfold dilution before quantification by HPLC. Additionally, samples of the diluted fortification controls were drawn off and passed through the Anatop 10 0.2 µm PTFE syringe filters used above, to check whether there was any sorption of the herbicide by the filters used in the extraction from media procedures described above. As the herbicide fortification solutions were made from the formulated products, some discrepancy from the calculated amount was expected due to allowable variation during manufacture of the formulated product. The actual amounts of the fortifications are presented in Table 4 along with the calculated values and showed the actual amount ranged from 90 to 122% of the of calculated. The actual fortification amounts were used to normalise all the degradation results, with all results being presented as percentages of the original fortification after correction for recovery. There was no detectable sorption of either atrazine or bromacil onto the Anatop 10, 0.2 µm PTFE syringe filters used during the clean-up of the extracted samples.

**Table 4:** Parameters for individual herbicides quantified by HPLC.

	Mobile phase	Retention time	Detection wavelength
Herbicide	(methanol:water, v/v)	(min)	(nm)
Atrazine	52:48	10.6	222
Bromacil	44:56	11.5	278

### Quantification by HPLC

In this study all sample analyses were run isocratically on a Shimadzu HPLC using a C-18, reverse-phase, 5 µm, 100 Å, 150 × 4.6 mm, ODS3 column (Prodigy, Phenomenex), a mobile phase flow rate of 1 mL/min and a column temperature of 35 °C. Sample injection volume was 20 µL. Quantification was by integration of peak areas. Calibration curves were made using a dilution series (0.05 to 50 ppm) of each herbicide prepared in methanol/water (50/50, v/v). Confirmation was by visual inspection of peak selection and base line and using external standards (1.0 and 10.0 mg/L) that were run after every 6–8 samples. The conditions for individual herbicides are presented in Table 4.

### Herbicide degradation profiles

As mentioned above, the principal cause of herbicide degradation is microbial degradation and in its general form follows a simple exponential decay model^15^, and is thus assumed to follow first-order kinetics (Eq. 1)^14^:

1 $${\text{d}}C/{\text{d}}t = - kC$$

where *C* is concentration in the soil (mg kg^−1^ soil), *t* is the time (days) and *k* is the degradation rate (days^−1^). Integration of Eq. (1) produces Eq. (2)

2 $$C_{t} = C_{0} \exp \left( { - kt} \right)$$

where *C*_*t*_ and *C*_0_ are concentrations (mg kg^−1^ soil) at times *t* and 0 respectively. The logarithmic form of Eq. (2) gives a linear relationship for *C* and *t* (Eq. 3)

3 $$\ln \left( {C_{t} } \right) = \ln \left( {C_{0} } \right){-}kt$$

The time taken for the concentration to be reduced to half the initial concentration is referred to as the half-life (t_½_ (days)). Substitution into Eq. (3) gives^14,46^:

4 $${\text{t}}_{\raise.5ex\hbox{$\scriptstyle 1$}\kern-.1em/ \kern-.15em\lower.25ex\hbox{$\scriptstyle 2$} } = \ln \left( 2 \right)/k$$

where *k* (the degradation rate) is the slope of the linear (logarithmic) plot. The common descriptor of degradation processes that fits first-order kinetics then is the half-life^14,47^. The time taken for the concentration of atrazine and bromacil to be reduced to half the initial concentration were calculated using Eq. (4). The standard error (SE) of the half-life is estimated by back prediction according to Eq. (5):

5 $${\text{SE}}\;\left( {{\text{t}}_{\raise.5ex\hbox{$\scriptstyle 1$}\kern-.1em/ \kern-.15em\lower.25ex\hbox{$\scriptstyle 2$} } } \right) = \left( {\ln \left( 2 \right) \cdot {\text{SE}}\left( {\text{k}} \right)} \right)/\left( {\text{k}} \right)^{2}$$

## Results

### Degradation of atrazine in four media under five different regimes

**Figure 1:**
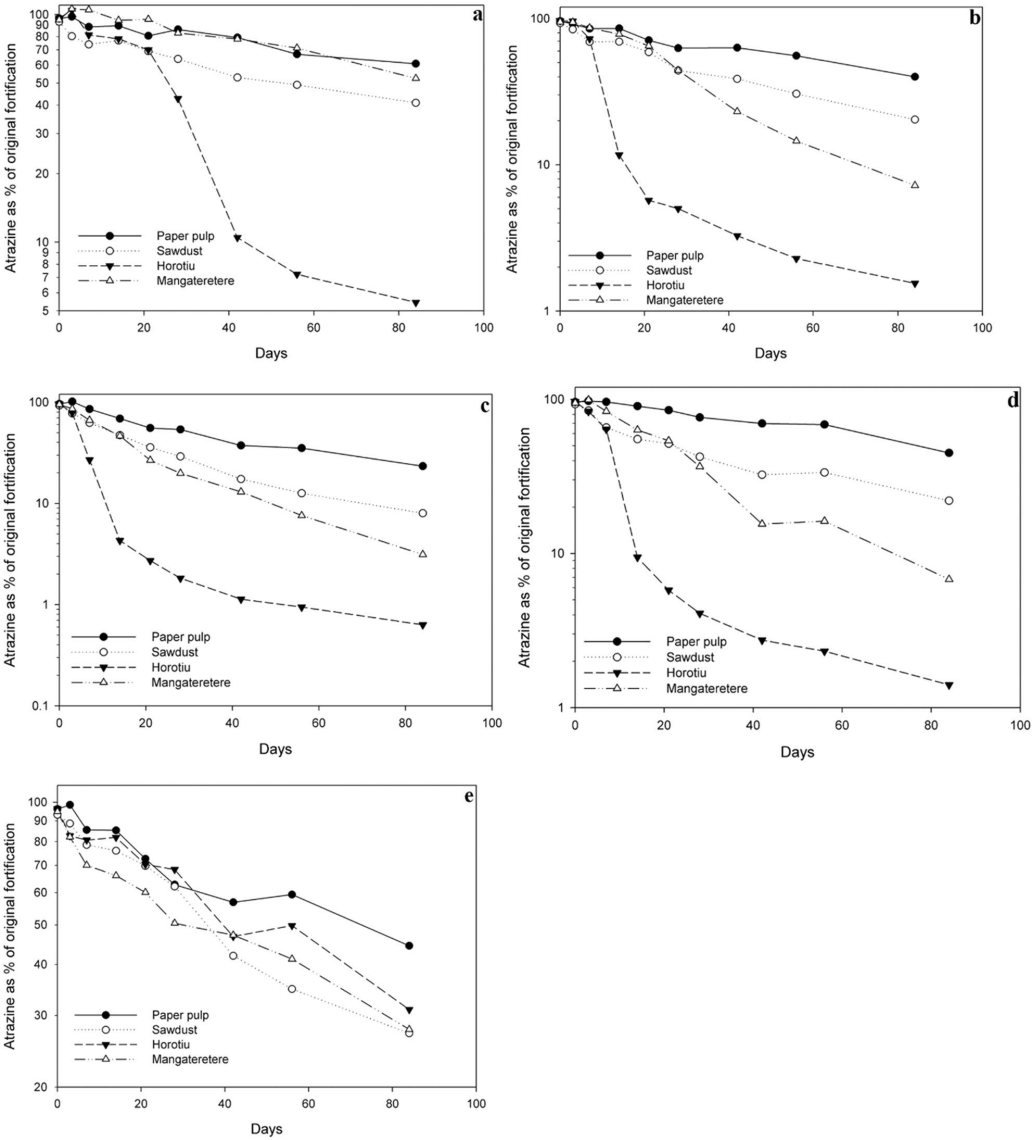
Atrazine degradation at (**a**) 10, (**b**) 20, (**c**) 30, (**d**) 20 ± 5, and (**e**) 20 °C sterilised with semi-log axis. Data points are average of duplicate samples.

The degradation of atrazine in all four media (wood pulp, sawdust, Horotiu soil and Mangateretere soil) at 10 °C, 20 °C, 30 °C, 20 ± 5 °C and at 20 °C in sterilised media are presented in Fig. 1a–e respectively. At 10 °C the degradation of atrazine was slow in wood pulp, sawdust and Mangateretere soil (t_½_ of 125, 75 and 89 d respectively) with > 40% of the original fortification remaining after 84 d. Degradation of atrazine in the Horotiu soil was considerably faster (t_½_ = 17 d) with > 90% of the original fortification degrading in just over 40 d. Semi-log plots of these reveal that for the wood pulp, sawdust and Mangateretere soil, degradation appears to closely follow first-order kinetics and linear regression shows that between 93.5 and 95.5% of the variation of the data is accounted for by the regression line (Table 3). For the Horotiu soil, linear regression can still explain 91.3% of the variation in the data yet a visual examination of the plot (Fig. 1a) reveals what appears to be a Logit (bi-sigmoidal) curve with faster initial degradation followed by a slower second phase.

Degradation of atrazine at 20 °C follows the same trend, however the half-lives are significantly shorter at this temperature (t_½_ of 70, 39 and 21 d for wood pulp, sawdust and Mangateretere soil respectively), and the differences in the degradation rates between the different media are more pronounced (Fig. 1b). The linear fit for first-order kinetics is very good for these three media with R^2^ values > 96% for each of them. However, degradation in the Horotiu soil is poorly described by first-order kinetics (R^2^ = 76.7%).

The plots of atrazine degradation at 30 °C (Fig. 1c) reveal a high degree of similarity to degradation at 20 °C, the only significant difference being the increased slope of the regression lines indicating more rapid degradation at 30 °C. Half-lives for wood pulp, sawdust and the Mangateretere soil were 39, 23 and 17 d respectively at this temperature. Again, degradation of atrazine in these three media is well described by first-order kinetics while the Horotiu soil shows greater deviation (R^2^ = 71.9%). Degradation of atrazine in the fluctuating temperature regime (Fig. 1d) is very similar to its degradation at 20 °C (Fig. 1b) which is the mean temperature of the fluctuating regime.

Figure 1e clearly shows that degradation of atrazine in the sterilised media is significantly reduced in all cases, although for wood pulp and sawdust the reduction is smaller (Table 3). This demonstrates the role of microbial activity on the degradation of this chemical. The most dramatic effect was on the Horotiu soil where the half-life of atrazine at 20 °C was increased from 13 to 55 d by sterilisation. Also, of note with this soil was that with sterilisation the degradation curve was now well described by first-order kinetics (Fig. 1e) with an R^2^ of 95.9%. Atrazine consistently underwent more rapid degradation in the non-sterilised Horotiu soil with the decay curve having the appearance of a logistic curve rather than an exponential curve.

### Degradation of bromacil in four media under five different regimes

**Figure 2:**
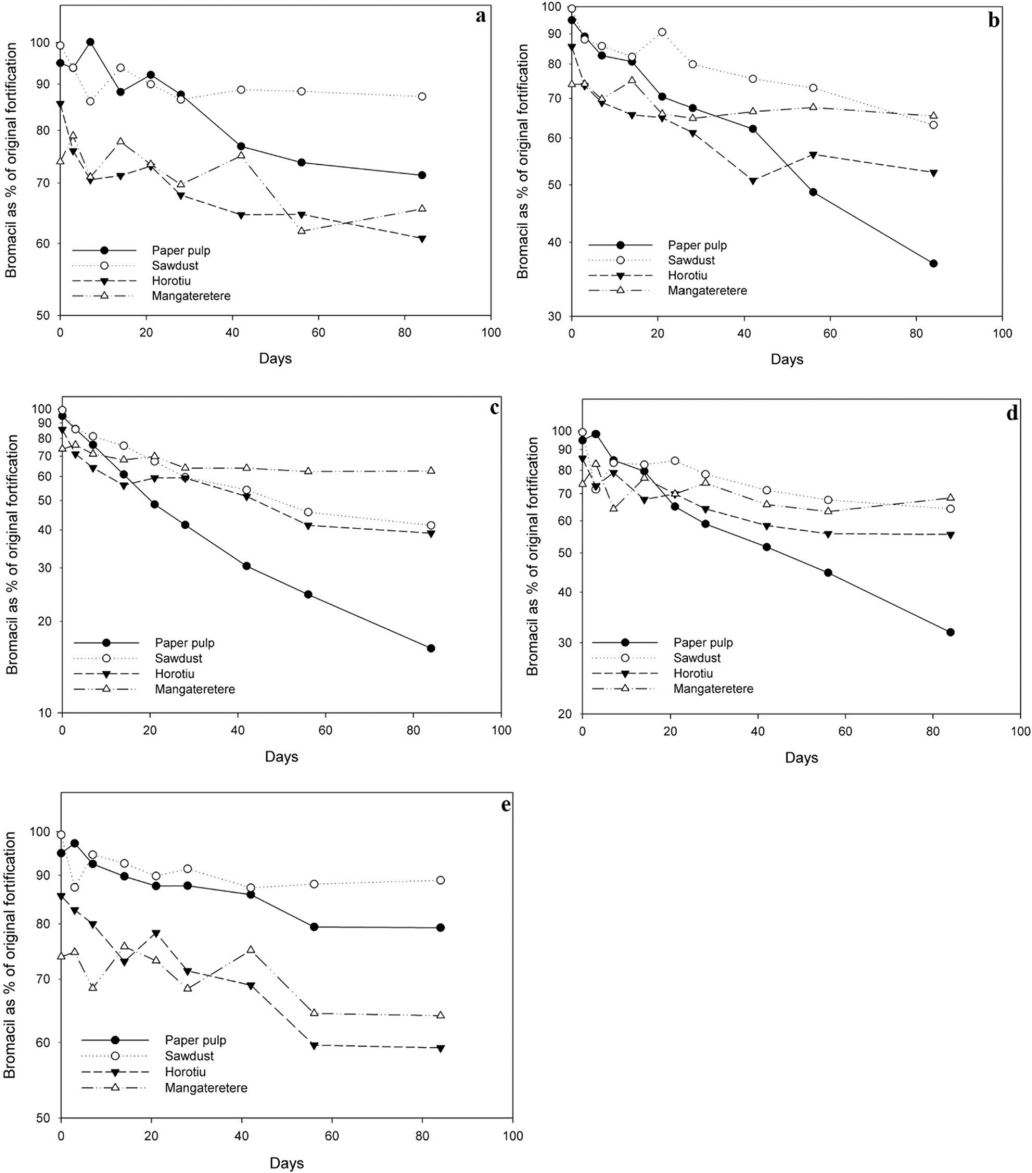
Bromacil degradation at (**a**) 10, (**b**) 20, (**c**) 30, (**d**) 20 ± 5, and (**e**) 20 °C sterilised with semi-log axis. Data points are average of duplicate samples.

The degradation of bromacil in all four media (wood pulp, sawdust, Horotiu soil and Mangateretere soil) at 10 °C, 20 °C, 30 °C, 20 ± 5 °C and at 20 °C in sterilised media are presented in Fig. 2a–e respectively. In these experiments the degradation of bromacil was considerably slower than that of atrazine except in wood pulp (Table 3). At 10 °C the degradation of bromacil was slow in all four media with > 60% of the original fortification remaining after 84 d (Fig. 2a). The considerable degree of extrapolation required resulted in large errors in the half-life estimates (t_½_ ranging from 170 to 724 d, (Table 3). This is reinforced by the low R^2^ values which reveal poor fits of the linear regression equation (Table 3). At 20 °C the degradation rate increased sufficiently in wood pulp (Fig. 2b), so that a realistic half-life (64 d) can be obtained with an R^2^ of 98.6% (Table 3). However, for sawdust, the Horotiu soil and particularly for the Mangateretere soil, the fit remains poor and the half-lives are well outside the duration of the experiments. Figure 2c shows that degradation of bromacil at 30 °C was significantly faster in wood pulp, sawdust and the Horotiu soil (t_½_ of 33, 68 and 85 d respectively) but remained largely untransformed in the Mangateretere soil (t_½_ = 300 d, Table 3).

As degradation was very slow in sawdust and the two soils, no valid comparisons can be made concerning enhanced degradation under the fluctuating temperature regime (Fig. 2d). However, degradation of bromacil in wood pulp was enhanced under the 20 ± 5 °C regime compared to that in the 20 °C regime (t_½_ of 52 and 64 d respectively). Also due to slow degradation, comparisons cannot be made for three of the sterile media (Fig. 2e). However, for wood pulp, sterilisation essentially stopped degradation (t_½_ of 64 and 288 d for the unsterilised and sterilised respectively).

## Discussion

The hypothesis to test with regard to herbicide degradation was that there was exponential decay which followed first-order kinetics. The test for this was a linear graph on a semi-log plot of the degradation data. Clearly, for many of the 40 degradation scenarios presented in Table 3 this hypothesis was proved true. Degradation of atrazine in either wood pulp, sawdust or the Mangateretere soil, at all temperature regimes, followed first-order kinetics. Degradation of atrazine in the Horotiu soil did not follow first-order kinetics at any temperature in field fresh soil. However, after the soil was sterilised, degradation of this herbicide did follow first-order kinetics.

### Degradation of atrazine and bromacil

In the case of bromacil, degradation rates were so slow that for many of the media/temperature combinations, neither degradation kinetics nor half-life could be accurately determined from the results of these 84 d experiments. The five cases where an accurate half-life could be determined (i.e. t_½_ < the duration of the experiment) all exhibited first-order kinetics. Thus, bromacil in wood pulp at 20, 30 and 20 ± 5 °C and in sawdust and in Horotiu soil at 30 °C underwent exponential decay. Unfortunately, for herbicides that exhibit very slow degradation, laboratory determinations of half-lives and other degradation parameters is not a viable proposition as soil microbial activity in the reaction flasks tends to diminish rapidly after ca. 3 months^48^.

In terms of relative rates of degradation in the four media, atrazine and bromacil exhibited nearly opposite trends. Using data from the 30 °C experiment, the relative rates of degradation of atrazine were Horotiu > Mangateretere > sawdust > wood pulp while for bromacil the ranking was wood pulp > sawdust > Horotiu > Mangateretere (Table 3). The most significant variation was for wood pulp where atrazine degraded more slowly compared to the other media while bromacil degraded more rapidly. The most likely reason for this is the pH of the wood pulp, which at 7.9 was the only basic medium. Bromacil being a very weak acid (pKa 9.27^49^) would be more soluble in the alkaline wood pulp and less soluble in the three acid media while atrazine being a weak base (pKa 1.70^49^) would be less soluble in the wood pulp and more soluble in the other media^50^. As the herbicide molecules must be in solution (bioaccessible) in order to be mineralised the pH mediated equilibria between solution and solid phase explains the relative rates of degradation for the two herbicides in the different media.

Mangateretere soil was the most acidic of the four media (Table 1). Therefore, atrazine would be more soluble in this soil than bromacil and their relative degradation rates in the four media will be the reverse of that for wood pulp. This is reflected in the results except for the Horotiu soil which initially exhibited faster degradation of atrazine. This was most probably due to enhanced microbial activity and not solubility effects^51^. The Horotiu soil and sawdust had similar pH levels, intermediate between the wood pulp and Mangateretere soil, so with the noted exception of atrazine in Horotiu soil, their degradation rates were also intermediate.

Since the degradation of both atrazine and bromacil follow first-order kinetics in these situations, the prediction and modelling of their environmental fate is easier as the degradation follows expected and well understood pathways^14,52,53^. Deviation from first-order kinetics may introduce considerable uncertainty in the estimated half-lives and degradation parameters, which in turn may result in discrepancies between predicted and observed dissipation patterns in the field^54^.

Two of the regimes under which degradation was evaluated were included specifically to investigate the role of the soil biota in degradation of the herbicides, atrazine and bromacil. The fluctuating temperature regime (20 ± 5 °C) was included to test whether the fluctuating temperature conditions that are normally found in the field are likely to impact on the degradation rate through the fluctuating temperature stimulating microbial activity^25^. Fluctuating temperature was postulated as the cause of enhanced degradation of clopyralid under similar regimes^24^. Conversely, the increase in degradation during the high temperature part of the cycle is thought unlikely to be completely offset by the reduced degradation during the cooler part of the cycle^55^. Determining degradation rate in the laboratory at a set temperature and assuming this will match degradation in the field when the average temperature is the same could lead to erroneous predictions^53^.

For the experiments reported here, the degradation half-lives for atrazine in Horotiu and Mangateretere soils (13 and 21 d, respectively) were identical in the 20 °C and the 20 ± 5 °C regimes while in the wood pulp and sawdust the half-lives for atrazine were slightly longer in the 20 ± 5 °C regime compared to the set temperature of 20 °C (Table 3). In the case of bromacil, due to its slow degradation, only a single comparison was possible, i.e. in wood pulp its degradation was significantly faster in the 20 ± 5 °C regime than at a constant 20 °C (52 and 64 d respectively). Thus, it would appear that the degradation of atrazine in fluctuating temperature conditions is less likely to deviate from predictions based on laboratory derived data than that of bromacil.

### Microbial degradation

The ultimate test for microbial degradation is to sterilise the soil and thus eliminate microbial activity from the equation^56,57^. In these experiments, sterilisation of the media produced mixed results. As the experiment was not maintained under strict sterile conditions, microbes could have been reintroduced when the flasks were opened to add water. However, it is highly unlikely that the introduced microbes would be in sufficient quantities or even be the required species to have any significant impact on the results. For atrazine, degradation half-lives remained nearly the same in both wood pulp and sawdust when the media was sterilised (Table 3) while the same process more than doubled its half-life in the two soils. For bromacil the only viable comparison that could be made was for wood pulp where sterilisation extended the half-life by a factor of five. Although these data are of dubious significance, it is interesting to note that in sawdust, bromacil experienced a similar extension of half-life (from 155 to 827 d) while the half-lives of this herbicide in the non-sterilised and sterilised regimes were similar for the two soils (Table 3).

It is evident from these results that microbial activity does play an important role in the degradation of atrazine as has previously been reported by many authors^7,57^. For bromacil, the evidence is less convincing but there is a common theme in the literature for microbial degradation of this herbicide, albeit very slow in some cases. Bromacil is variously recognised as less biodegradable^58^, while Wolf & Martin^56^ and Zimdahl et al.^37^ make a strong case for its microbial degradation. More recently, organisms that use this chemical as their sole source of C and N have been isolated^59^. The results from the experiments reported here support the importance of microbial activity in the degradation of bromacil in both the wood pulp and sawdust, where half-lives were significantly longer in the sterilised media. However, half-lives in the sterilised soils were the same as for the non-sterilised soils indicating that other degradation processes such as chemical hydrolysis were predominant.

### Degradation half-life

It is generally accepted the principal cause of herbicide degradation is microbial degradation and this usually follows first-order kinetics with an exponential decay curve. Thus, in examining herbicide degradation the first hypothesis to test is that herbicide degradation is exponential^55^. The test for this is to plot each decay curve on a semi-log scale and test for a straight line (linear regression analysis)^14,53^. For atrazine the range of field half-lives reported in the United States Agricultural Research Service (ARS-USDA) pesticide properties database range from 18 to 148 d with an average of 60 d^60^. The low half-life of 18 d is from the GLEAMS user manual^61^, and its origins are uncertain. The next lowest half-lives are several in the range of 45–50 d. In New Zealand Rahman & Brown^62^, using bioassay methods to determine atrazine residues in samples collected from field trials, found half-lives of 56–112 d and 210–300 d for a Hamilton silt loam and a Matawhero heavy silt loam respectively, while Rahman et al.^63^ determined a half-life of about 90 days for both a Horotiu sandy loam soil and a Hamilton clay loam soil using similar methods. In a laboratory experiment Ghani et al.^64^ determined a dissipation time (DT_50_) of 45–55 d for a Horotiu sandy loam soil at 22.5 °C.

The half-lives found in the experiments reported here generally relate well to both the international and local findings. Degradation of atrazine in the Horotiu soil was much faster than that reported internationally, and it did not conform to the simple first-order kinetics model. Degradation in the Mangateretere soil was also relatively fast but did follow first-order kinetics and can be compared to field data for other New Zealand soils^63^. Considering the field degradation in the Hamilton soil would have occurred while soil temperatures ranged between 10 and 20 °C, then degradation in Mangateretere soil is not dissimilar to that in the Hamilton soil^63^. However, degradation in the Matawhero soil was considerably slower than any of the laboratory determined rates^63^. Degradation of atrazine in the wood pulp and sawdust, although slower than in the two New Zealand soils were quite comparable to some of the rates reported by Wauchope et al.^60^.

There are few data reported for half-life of bromacil in New Zealand where it is used mostly for weed control in asparagus^65^. Sanders et al.^65^ reported detectable residues have generally dissipated within 3–6 months. This could indicate a half-life of less than 60 days or dissipation could be accounted for by the herbicide leaching rather than degrading. As they also report high concentrations of bromacil at greater depths it is probably the latter which is a better description of the reason for dissipation of bromacil from the surface soil layer.

The great majority of the data for bromacil comes from USA where it is used extensively for weed control in citrus orchards in Florida and California and in pineapples in Hawaii^66,67^. In a field study on a silt loam soil Gardiner et al.^68^ determined a half-life of 5–6 months for bromacil when applied at 4.48 kg a.i./ha. Using an undescribed loam soil Zimdahl et al.^37^ showed that degradation of bromacil followed first-order kinetics with half-lives of 214 and 158 d at 13.2 and 31.2 °C respectively. In a 125 day experiment using a flooded sandy soil (pH 7.1 and OC 1.1%), Wolf and Martin^56^ found bromacil to have a half-life of 146 d and that degradation essentially ceased when the soil was sterilised. They also postulated that degradation of bromacil in their experiments followed a hyperbolic type of rate law, with degradation over the first 10 days much greater than for the following 105 days. However, this was based solely on the first two bromacil quantifications carried out on Days 0 and 10 of the study and more likely reflects the difficulty in extraction of residues aged for even a short time^69^ especially as they were using only a weak extractant (ethanol).

In a study using^14^ CO_2_ evolution as the means of determining bromacil degradation in a sandy soil (pH 7.3, OC 0.7%) and a mucky peat (pH 5.4, OC 48%) at three temperatures, Madhun & Freed^58^ claimed there was little difference in degradation rates due to soil type but that temperature had a major effect, with half-lives ranging from 46,200 ± 38,707 d at 25 °C to 5,856 ± 3,957 d at 35 °C. But as these results were extrapolated from a 27 d and a 36 d experiment for the sandy soil and mucky peat respectively they appear to be unreliable and contrary to field observations. The half-lives reported in the SCS/ARS/CES pesticide properties database^60^ derived from a variety of sources including the manufacturer (E.I. du Pont de Nemours and Co.) range from 61 to 349 d with an average of 207 d.

The half-lives for bromacil determined in the experiments reported here are comparable to those reported in the literature. The average half-life in wood pulp of 64 d at 20 °C corresponds well with the shortest half-life of 61 d reported in the literature. The half-lives of 136 d and 155 d for Horotiu soil and sawdust respectively are shorter than the average of 207 d in the ARS-USDA database but still well within the range of results. It is likely the fast dissipation in our sandy soils has allowed growers to use bromacil in asparagus crops. In most other countries the less persistent analogue terbacil is the major herbicide used in asparagus^20^. The long persistence in the Mangateretere soil (t_½_ = 485 d) appears to be greater than the longest reported in the ARS-USDA database (349 d) but, considering the large extrapolation error, the findings are not significantly different from those reported in the database.

### Causes of enhanced degradation

In New Zealand enhanced degradation of a herbicide was first reported for EPTC in 1979^70^, and an *Anthrobacter* sp. bacteria was later identified as being responsible^71^. Rahman et al.^72^ in a study on the degradation of flumetsulam reported the rate of degradation over the first 2 weeks as being considerably faster than in subsequent weeks, while James et al.^10^ reported rapid initial dissipation of chlorsulfuron and triasulfuron which resulted in dissipation times (DT_50_) considerably shorter than the calculated half-lives (t_½_) when first-order kinetics were assumed. In these experiments the period of rapid degradation was about 3 days and the half-lives were greater than the dissipation times by factors of 3–7 in the laboratory and 1.5–2 for field determined rates. Bolan & Baskaran^73^ found enhanced degradation of the herbicide 2,4-D by soil microbial activity particularly in soils containing greater than 12% organic carbon. More recently, satisfactory modelling of the field dissipation of acetochlor and terbuthylazine using a range of predictive simulation models could only be achieved when biphasic, two-rate or two-compartment sub-models were invoked^11,74^. Similarly for predictions of field persistence of atrazine in a Bruntwood silt loam soil (very similar to the Horotiu soil we used) using the model Opus2, Müller et al.^52^ required the implementation of a sigmoidal degradation equation to explain the accelerated degradation observed in the field.

For both atrazine and bromacil a gram-negative rod bacterium (*Pseudomonas* sp.) has been isolated from atrazine and bromacil-contaminated soil and used to demonstrate its ability to degrade these chemicals at faster than normal rates^59,75^. In fortified soil inoculated with the active strains of this species, results showed that 50% of the atrazine fortification was mineralised in 14 days^75^ while bromacil was completely degraded within 7 days and a molar equivalent amount of bromide was recovered^59^. In New Zealand Aislabie et al.^76^ isolated the bacterium *Anthrobacter nicotinovorans* from a Himatangi soil exhibiting enhanced rates of atrazine mineralisation.

## Conclusions

The hypothesis being tested in this research was that herbicides degrade more rapidly in the presence of high organic matter soil amendments. The reasoning behind this was that herbicides tend to degrade more rapidly in high organic matter soils due to this supporting greater microbial biomass, and therefore high organic matter waste material would simply be an extension of this. However, it was found that the pH of the media had a more profound effect on the degradation rate than the organic carbon content. Microbial degradation also proved to be an important factor in the mineralisation of these herbicides and was the cause of enhanced degradation in the Horotiu soil which had a long history of application of atrazine. Removal of the microbial component by sterilisation slowed the degradation of atrazine in the two soils more than it did in the two organic media. For bromacil the converse was true where sterilisation of the organic media had a significant impact in slowing degradation while it had no effect on degradation in the two soils. Therefore, it can be concluded that the hypothesis is not correct because organic matter content per se did not directly relate to degradation rates which were mainly governed by pH and microbial activity.

Although an increase in the percentage of organic carbon in the soil can often affect herbicide activity in soil by increasing adsorption sites and providing better conditions for rapid microbial activity, this relates to more highly decomposed organic matter than what was provided by the sawdust and wood pulp used here. Previously, we showed that organic carbon content plays an important role in controlling herbicide sorption in the forestry waste products, sawdust and wood pulp^26^. We concluded that the structural differences of the organic carbon are very important. That is the larger particle size of sawdust and its related reduced surface area to weight ratio play a significant role in the different behaviours of the herbicides in the high organic carbon media^26^. In this research, we also demonstrated that the persistence of herbicides in the forestry waste products did not correlate to the level of organic carbon contents, but the pH and microbial activity of the media had more profound effects on the desorption and subsequent degradation rate of herbicides in sawdust and wood pulp. These results also indicate that the diverse characteristics of the two forestry waste products frequently had greater impacts on the behaviour of herbicides than did their organic carbon content alone. This has important ramifications for the use of these waste products in agriculture. Thus incorporation of sawdust and wood pulp into soil as part of a system to get rid of these waste materials should not cause the major increases in rates of herbicide degradation that are suggested by most work involving soil with high organic matter content, though this may change after many years as the material decomposes to provide greater surface area of organic matter and increased microbial populations.
